# Quantitative CT-derived lobar inter-tapering as a candidate imaging feature associated with airway-predominant non-emphysematous COPD

**DOI:** 10.3389/fmed.2026.1876551

**Published:** 2026-09-07

**Authors:** Haibo Bian, Yiting Xiao, Yang Cheng, Jingru Zhang, Guopeng Shi, Yongai Li, Ruina Jin, Yan Li, Zhanyu Shi, Yangyang Xu, Xiazhen Wang, Wanping Wang

**Affiliations:** 1 Department of Respiratory and Critical Care Medicine, Changzhi People's Hospital, The Affiliated Hospital of Shanxi Medical University, Changzhi, China; 2 Changzhi People's Hospital, The Affiliated Hospital of Shanxi Medical University, Changzhi, China; 3 Changzhi People's Hospital, The Affiliated Hospital of Changzhi Medical College, Changzhi, China; 4 Department of Radiology, Changzhi People's Hospital, The Affiliated Hospital of Changzhi Medical College, Changzhi, China; 5 Beijing Key Laboratory for Design and Evaluation Technology of Advanced Implantable & Interventional Medical Devices, Beijing Advanced Innovation Center for Biomedical Engineering, School of Biological Science and Medical Engineering, Beihang University, Beijing, China

**Keywords:** airway morphology, chronic obstructive pulmonary disease, inter-tapering, non-emphysematous COPD, quantitative computed tomography

## Abstract

**Background:**

Chronic obstructive pulmonary disease (COPD) is a heterogeneous disorder involving varying contributions from airway abnormalities and emphysematous destruction. However, structural airway alterations in patients with airflow limitation but minimal emphysema remain incompletely characterized. Quantitative CT (QCT)-based airway morphology analysis may capture additional aspects of airway geometry associated with airway-predominant structural patterns.

**Objectives:**

This study aimed to evaluate lobar airway inter-tapering abnormalities on quantitative CT in patients with non-emphysematous COPD (NE-COPD) and investigate whether incorporation of inter-tapering features improves discrimination within the studied cohort.

**Methods:**

This retrospective cross-sectional study included 173 participants who underwent inspiratory thin-section chest CT and post-bronchodilator pulmonary function testing between January 2023 and March 2025. NE-COPD was defined as airflow limitation with minimal emphysema quantified by low-attenuation area percentage below −950 HU (LAA%-950 < 5%). Automated airway segmentation and centerline-based analysis were used to quantify fourth-generation lobar inter-tapering indices. A clinical model incorporating age, sex, and smoking exposure was constructed. An imaging-augmented model was subsequently developed using LASSO-selected lobar inter-tapering features together with global airway branching complexity. Model performance was evaluated using receiver operating characteristic analysis, bootstrap validation, calibration assessment, decision curve analysis, and SHAP interpretation.

**Results:**

Among the 173 participants, 87 had NE-COPD and 86 were Non-COPD controls. Compared with Non-COPD controls, patients with NE-COPD demonstrated altered fourth-generation lobar inter-tapering patterns in multiple lobes. The clinical model achieved an area under the curve (AUC) of 0.717. After incorporation of quantitative airway features, the imaging-augmented model demonstrated improved discrimination, with an AUC of 0.851. Bootstrap validation demonstrated stable model performance. SHAP analysis indicated that four lobar fourth-generation inter-tapering indices accounted for a substantial proportion of feature contribution within the fitted model. Decision curve analysis suggested higher theoretical net benefit of the imaging-augmented model within the evaluated threshold probability range.

**Conclusion:**

Quantitative CT-derived lobar inter-tapering was associated with pulmonary function-defined non-emphysematous COPD. These findings suggest that lobar inter-tapering represents a candidate geometric CT descriptor for characterizing airway-predominant structural patterns. Further multicenter studies with external validation and integration of complementary quantitative CT descriptors are required to determine its broader clinical utility.

## 1 Introduction

Chronic obstructive pulmonary disease (COPD) is a heterogeneous respiratory disorder characterised by persistent airflow limitation and variable contributions from airway disease and parenchymal destruction (1). Chest computed tomography (CT) has an established role in evaluating emphysema, airway abnormalities, and CT-defined structural patterns (2–4). However, a substantial proportion of patients with spirometrically confirmed COPD show minimal or no emphysematous destruction on CT. In these individuals, pathological changes may be predominantly related to airway structural alterations and small airway abnormalities rather than visible parenchymal destruction, making subtle structural abnormalities difficult to detect by routine visual assessment (5–7). Therefore, quantitative imaging markers capable of capturing airway-predominant structural alterations may provide additional information in this subgroup.

Small airway disease is increasingly recognised as a central component of COPD pathogenesis and may contribute to airflow limitation before extensive emphysematous change becomes apparent (5–7). Conventional CT interpretation primarily focuses on visually apparent abnormalities, including emphysema, bronchial wall thickening, bronchiectasis, and air trapping. Although these findings are clinically valuable, they may not fully capture subtle alterations in airway calibre transitions across generations, particularly in patients with non-emphysematous COPD (NE-COPD). Quantitative CT (QCT) has therefore become an important tool for objective assessment of airway morphology and COPD phenotyping (3, 4, 8, 9).

Large-scale COPD imaging cohorts have demonstrated substantial heterogeneity in CT-defined structural patterns. The COPDGene study established a comprehensive framework for evaluating genetic, clinical, and imaging characteristics of COPD, including emphysema distribution, airway abnormalities, and disease progression (3, 4, 10, 11). Similarly, the SPIROMICS cohort developed standardized multicenter quantitative CT protocols for assessing emphysema, air trapping, and airway structural characteristics (12, 13). The Fleischner Society statement further emphasized that COPD comprises multiple CT-defined subtypes involving varying contributions from emphysema, airway disease, and small airway dysfunction (4, 12). More recent multimodal studies have demonstrated that smokers with COPD may exhibit distinct combinations of emphysema burden, parametric response mapping (PRM)-defined functional small airway disease, gas trapping, and airway morphology, highlighting the need for additional quantitative approaches to characterize different dimensions of COPD-related airway and parenchymal abnormalities (12, 14).

Previous quantitative CT studies have primarily focused on specific dimensions of airway abnormalities, including airway wall thickness, Pi10, airway lumen measurements, and airway count, which provide important information regarding airway remodeling, narrowing, and structural loss (15, 16). Functional imaging approaches, such as expiratory CT-derived gas trapping and parametric response mapping (PRM), have further expanded the ability to characterize functional small-airway abnormalities (12, 14). However, these established descriptors mainly describe individual structural or functional components of COPD, whereas the geometric organization of airway branching across generations remains relatively less explored. Inter-tapering quantifies the relative reduction in airway calibre from proximal parent airways to distal daughter branches and therefore represents a descriptor of airway branching geometry rather than a direct measure of a specific pathological process (17). Advances in automated airway segmentation and generation-specific airway analysis now allow detailed evaluation of regional airway geometry across the bronchial tree (18). However, whether lobar and generation-specific inter-tapering abnormalities are associated with spirometric COPD in individuals with minimal emphysematous destruction remains unclear.

We hypothesised that quantitative CT-derived lobar inter-tapering would differ between individuals with NE-COPD and Non-COPD controls and would be associated with airflow limitation. Therefore, this study aimed to compare fourth-generation lobar inter-tapering between patients with spirometric COPD without emphysema and Non-COPD controls, evaluate its relationship with pulmonary function, and determine whether incorporation of inter-tapering metrics into clinical variables improves discrimination of NE-COPD. Within the broader framework of quantitative COPD imaging, generation-specific airway geometry represents a relatively underexplored dimension of airway organization. Accordingly, this study was designed to evaluate whether inter-tapering could serve as a candidate geometric CT descriptor associated with airway-predominant COPD features, while recognizing that its incremental value beyond established quantitative CT descriptors requires further investigation.

## 2 Materials and methods

### 2.1 Study design and participants

This retrospective cross-sectional study was conducted at Changzhi People's Hospital. The study protocol was approved by the institutional ethics committee (Approval No. 2025K056), and the requirement for written informed consent was waived due to the retrospective nature of the study. Adults who underwent both volumetric inspiratory thin-section chest CT and post-bronchodilator pulmonary function testing between January 2023 and March 2025 were retrospectively screened. These examinations were performed as part of routine clinical evaluation according to physician discretion, including assessment of respiratory symptoms, suspected or established respiratory disease, and evaluation of abnormal clinical findings.

**Figure 1:**
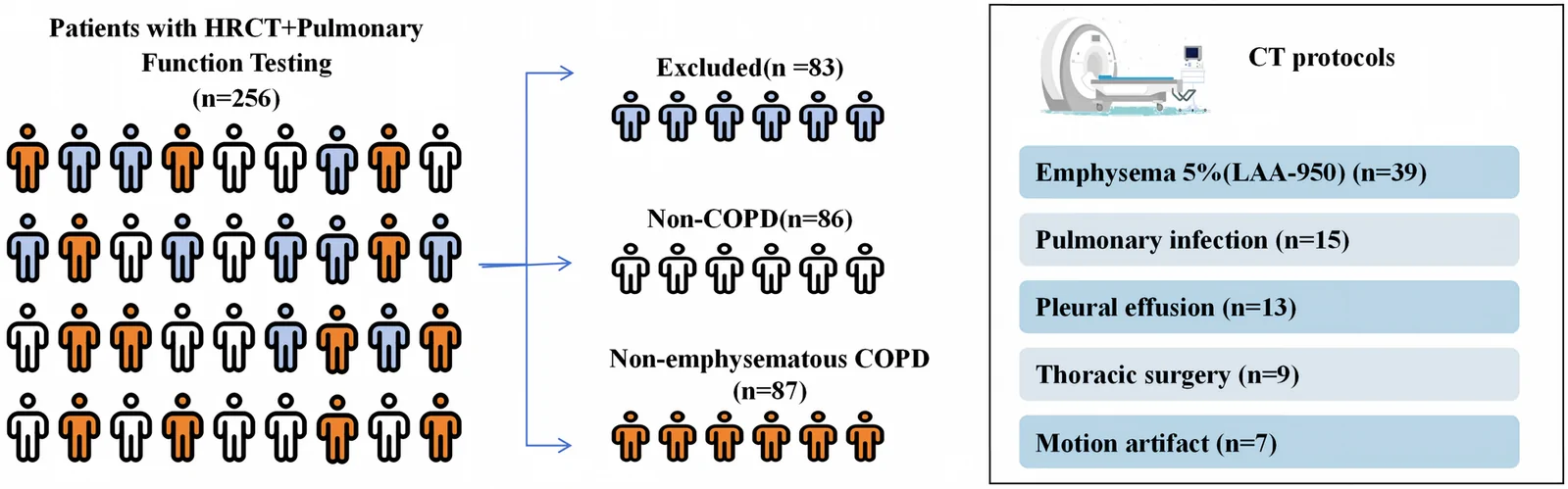
Flowchart of patient selection and study cohort formation.

The inclusion criteria were as follows: age ≥18 years, availability of inspiratory thin-section chest CT images with adequate image quality, and availability of post-bronchodilator spirometry performed within an appropriate clinical interval. Patients were excluded if they had a history of lung resection, extensive pulmonary fibrosis, active pulmonary infection, large pulmonary lesions, or other diseases causing substantial distortion of bronchial anatomy, as well as severe motion artifacts, incomplete CT datasets, or unavailable or poor-quality pulmonary function measurements. The final study population consisted of 173 participants, including 87 patients with non-emphysematous COPD (NE-COPD) and 86 Non-COPD controls. A detailed participant selection flow diagram is provided in Figure 1. Because all participants underwent CT and pulmonary function testing as part of clinical evaluation, and CT examinations were performed 24–48 h prior to pulmonary function testing, the Non-COPD group should be interpreted as a clinical comparison population rather than a population-based healthy cohort.

### 2.2 Definition of NE-COPD and emphysema assessment

COPD was defined according to post-bronchodilator spirometry criteria with a forced expiratory volume in one second to forced vital capacity ratio (FEV₁/FVC) < 0.70, consistent with commonly used clinical diagnostic criteria (1). Non-emphysematous COPD (NE-COPD) was defined as the presence of spirometric airflow limitation accompanied by minimal emphysematous destruction on quantitative CT. Emphysema burden was quantified using the percentage of low-attenuation areas below −950 HU on inspiratory CT (LAA%-950) (15, 19). Participants with LAA%-950 <5% were classified as having minimal emphysema and were eligible for inclusion in the NE-COPD group. This threshold was selected based on previous quantitative CT studies that commonly use LAA%-950 to identify individuals with minimal or absent emphysematous destruction (20). Because the present study focused on airway-predominant structural abnormalities, quantitative emphysema assessment rather than subjective visual grading was used for phenotype definition.

### 2.3 CT acquisition and image reconstruction

**Figure 2:**
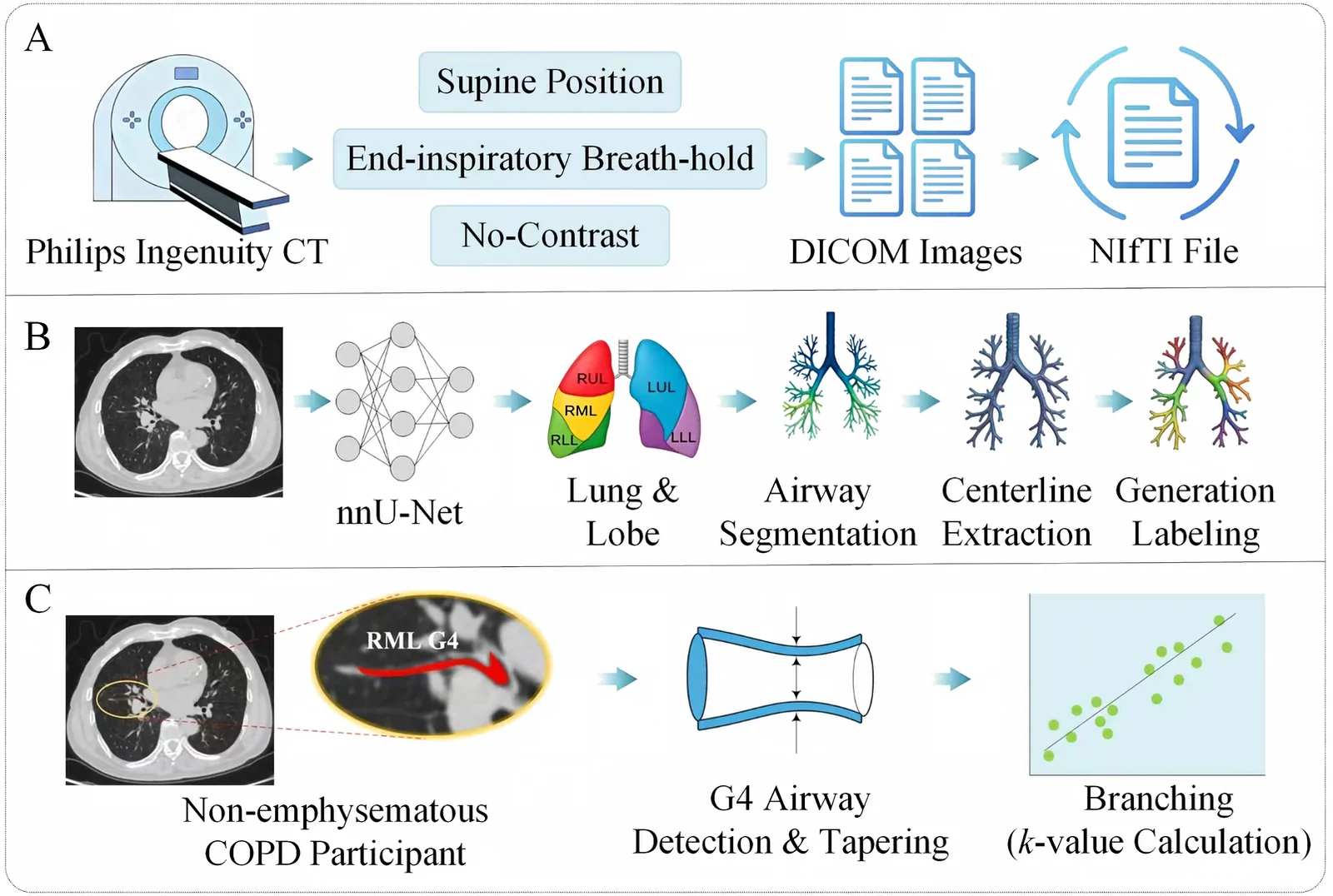
Workflow of automated quantitative airway analysis. **(A)** CT acquisition and image conversion. **(B)** Deep-learning-based lung, lobe, and airway segmentation with centerline extraction and generation labeling. **(C)** Measurement of fourth-generation right middle lobe inter-tapering and global airway branching complexity.

All participants underwent non-contrast chest CT in the supine position during end-inspiratory breath-hold using the same CT scanner (Philips Ingenuity CT). Tube voltage was 120 kVp; helical acquisition used a rotation time of 0.5 s, pitch of 1.0, detector collimation of 0.625 mm, and reconstruction with a 512  ×  512 matrix, B kernel, 1.0 mm slice thickness, and 1.0 mm interslice spacing. Images were reconstructed using a high-resolution reconstruction algorithm and subsequently transferred to a dedicated workstation for quantitative airway analysis (Figure 2A).

### 2.4 Automated airway segmentation and quantitative airway analysis

**Figure 3:**
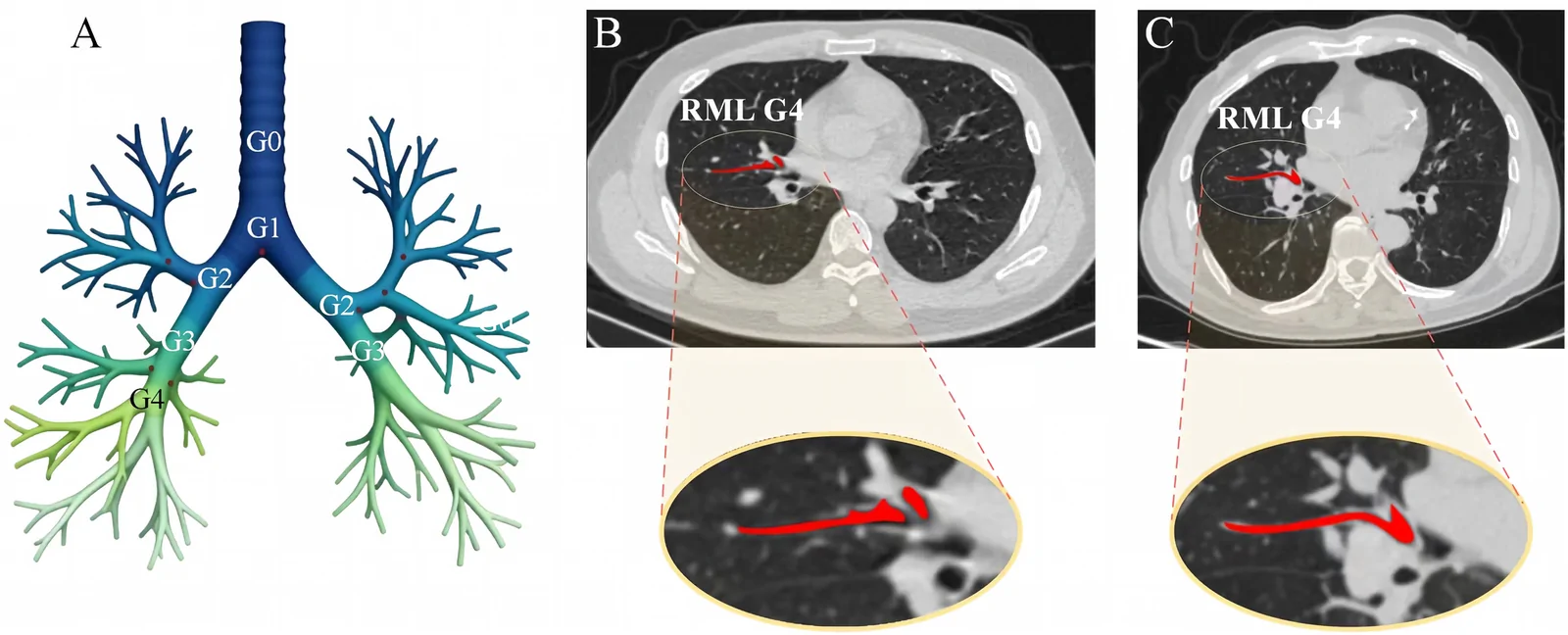
Representative chest CT images of a Non-COPD control and a patient with NE-COPD. **(A)** Axial chest CT image of a Non-COPD; **(B)** Axial chest CT image of a patient with NE-COPD; **(C)** The location of the fourth-generation airway in the right middle lobe (RML G4) is indicated in red in both panels.

**Figure 4:**
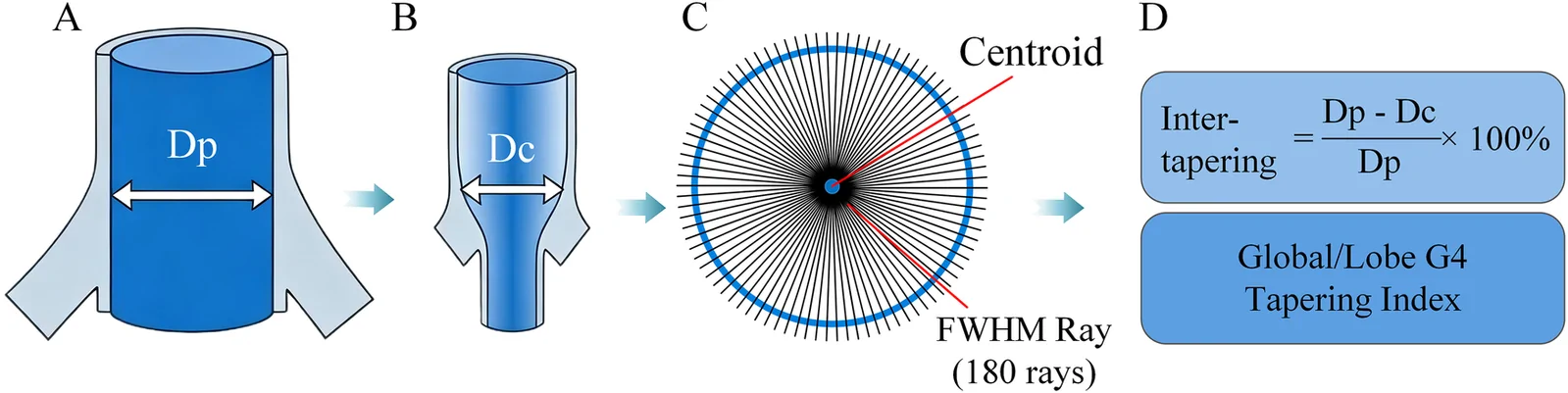
Definition and calculation of the fourth-generation airway inter-tapering index. Illustration of the airway inter-tapering measurement procedure. **(A)** Definition of the parent airway diameter (Dp). **(B)** Definition of the child airway diameter (Dc) at the subsequent airway branch. **(C)** Airway lumen cross-section was detected using a centroid-based full-width-at-half-maximum (FWHM) method with 180 radial rays. **(D)** Airway inter-tapering was calculated as (Dp−Dc)/Dp × 100%, and lobe-specific fourth-generation (G4) airway inter-tapering indices were subsequently derived. Higher values indicate a greater relative decrease in airway caliber from the parent to the daughter airway.

Automated airway segmentation results were reviewed by trained observers. Cases with incomplete airway tracking, severe segmentation artifacts, incorrect airway topology, or unreliable fourth-generation airway assignment were corrected when feasible or excluded from quantitative analysis (Ten cases required manual correction and two cases were excluded due to inadequate airway segmentation quality. The average measurable fourth-generation airway counts per lobe were: LLL 3.98, LUL 2.11, RLL 2.02, RML 2.02, and RUL 4.41). Quantitative measurements were performed only on airway segments passing quality assessment. The airway analysis pipeline consisted of automated lung and airway segmentation, airway centerline extraction, airway generation assignment, and quantitative measurement of airway calibre (Figure 2B). The lung and airway structures were initially segmented using deep-learning-based methods incorporating TotalSegmentator and nnU-Net frameworks (21, 22). The airway tree was subsequently processed using centerline thinning algorithms to obtain airway trajectories and branching structures (23). Airway generations were assigned according to sequential branching order, and fourth-generation airway segments were selected for generation-specific analysis (Figures 2C, 3). The fourth-generation level was selected because it provided a balance between measurable airway representation and avoidance of excessive segmentation uncertainty in more distal branches. For each airway branch, lumen diameter was measured along the centerline. TO reduce the influence of extreme measurements caused by partial-volume effects, segmentation uncertainty, or irregular airway geometry, the 10% trimmed mean was used to summarize airway diameter measurements within each generation. The inter-tapering index was calculated to quantify airway calibre transition between parent and daughter airway branches using the following equation:

$$\mathrm{Inter}\text{-}\mathrm{tapering}\;\mathrm{index}=\left[\frac{\left(\mathrm{Dp}\text{-}\mathrm{Dc}\right)}{\mathrm{Dp}}\right]\times 100\text{\%}$$

where D represents the mean airway diameter (Figure 4). A higher inter-tapering value indicates greater calibre reduction between sequential airway generations and represents a quantitative descriptor of airway calibre transition (17, 24). All automated segmentation results, centerline reconstructions, and airway generation assignments underwent visual quality control by trained observers blinded to pulmonary function results. Cases with obvious segmentation failure, incorrect airway topology, or unreliable generation assignment were corrected when possible; cases that could not be reliably corrected were excluded before quantitative analysis. In addition to lobar inter-tapering, a global airway branching index was calculated to describe overall airway branching complexity. For each participant, the number of airway segments in each generation was recorded from generation 0 to generation 14 (Figure 2B). Airway generation number was modelled as a function of the natural logarithm of airway segment count using second-order polynomial regression. The coefficient representing the rate of change in branching complexity across generations was defined as the global k value. A less negative k value indicates altered global branching complexity based on the distribution of detectable airway segments across generations.

### 2.5 Pulmonary function testing

Spirometry was performed according to the American Thoracic Society and European Respiratory Society recommendations (25). Measurements included forced vital capacity (FVC), forced expiratory volume in one second (FEV₁), FEV_1_/FVC ratio, and forced expiratory flow between 25% and 75% of FVC (FEF25%–75%). All airflow measurements were obtained after bronchodilator administration. Smoking exposure was quantified using pack-years, calculated as the number of cigarettes smoked per day divided by 20 and multiplied by the number of smoking years. Never-smokers were assigned a value of zero pack-years.

### 2.6 Statistical analysis

All statistical analyses were performed using R software (version 4.5.1). Continuous variables were evaluated for distribution characteristics. Normally distributed variables were presented as mean ± standard deviation, whereas non-normally distributed variables were summarized as median with interquartile range. Categorical variables were presented as numbers and percentages. Continuous variables were summarized as mean ± standard deviation or median (interquartile range), according to data distribution. Between-group comparisons were performed using Student's *t*-test or Mann–Whitney *U*-test for continuous variables and chi-square test or Fisher's exact test for categorical variables, as appropriate. Associations between quantitative airway metrics and pulmonary function parameters were assessed using Spearman correlation analysis.

### 2.7 Construction of clinical and imaging-augmented models

A clinical model was constructed using clinically relevant baseline variables, including age, sex, and smoking exposure. An imaging-augmented model was subsequently developed by incorporating quantitative airway features selected through the feature selection procedure. The final imaging-augmented model consisted of clinical variables (age, sex, and smoking exposure), global airway branching complexity (global k value), and four LASSO-selected fourth-generation lobar inter-tapering indices (G4 LLL, G4 LUL, G4 RML, and G4 RUL). All candidate quantitative airway metrics were entered into the LASSO procedure together with clinically relevant variables to identify predictors for the final model. To reduce dimensionality and avoid overfitting, least absolute shrinkage and selection operator (LASSO) logistic regression was performed. The optimal lambda value selected by 10-fold cross-validation was 0.05964653. Variables retained at this lambda value were entered into the final multivariable logistic regression model (26). Model discrimination was evaluated using the area under the receiver operating characteristic curve (AUC), sensitivity, and specificity. Comparisons between models were performed using DeLong's test (27).

### 2.8 Model validation and interpretation

Internal validation was performed using 1,000 bootstrap resampling iterations. The complete model development process, including feature selection and model refitting, was repeated within each bootstrap sample to estimate optimism and obtain optimism-corrected performance estimates. Apparent performance, bootstrap optimism, and optimism-corrected performance were reported to provide a comprehensive assessment of internal validity. Model calibration was evaluated using calibration plots and quantified by calculating the calibration slope and calibration intercept. Clinical utility was assessed using decision curve analysis (DCA), with net benefit estimated across a range of threshold probabilities to evaluate the potential value of applying the prediction models (28). To interpret the contribution of individual imaging features, SHAP (Shapley Additive Explanations) analysis was performed for the final imaging-augmented model. A two-sided *P* value < 0.05 was considered statistically significant (29, 30).

## 3 Results

### 3.1 Participant characteristics

A total of 173 participants were included in the final analysis, comprising 87 patients with NE-COPD and 86 Non-COPD controls. The participant selection process is summarized in Figure 1. Baseline characteristics are presented in Table 1. Patients with NE-COPD were older than controls (60.18 ± 9.65 years vs. 53.29 ± 11.86 years, *p* < 0.001), and the proportion of men was higher in the COPD group (71.3% vs. 51.2%, *p* = 0.011). Smoking history was also more common in patients with COPD than in controls (37.9% vs. 22.1%, *p* = 0.035), and the smoking index was higher in the COPD group (*p* < 0.001). Body mass index and body weight did not differ significantly between groups. Patients with NE-COPD had lower airflow-related pulmonary function indices than Non-COPD controls. Compared with Non-COPD controls, patients with NE-COPD had lower FEV₁, FEV₁/FVC, peak expiratory flow, MEF75%, MEF50%, MEF25%, FEF25%–75%, and maximal voluntary ventilation, all with *p* < 0.001. FVC did not differ significantly between groups (*p* = 0.396) (Table 1). Because age, sex, and smoking exposure differed between groups, these variables were considered potential confounding factors and were incorporated into subsequent multivariable analyses.

**Table 1:** Baseline characteristics and pulmonary function of the study population.

Variable	Non-COPD (*n* = 86)	NE-COPD (*n* = 87)	*p* value
Age (years)	53.29 ± 11.86	60.18 ± 9.65	<0.001
Sex, No. (%)			0.011
Male	44 (51.2)	62 (71.3)	
Female	42 (48.8)	25 (28.7)	
BMI (kg/m²)	25.17 ± 3.56	25.01 ± 3.65	0.800
Smoking history, No. (%)			0.035
Never	67 (77.9)	54 (62.1)	
Former or current	19 (22.1)	33 (37.9)	
Smoking index, median (IQR)	0 (0-0)	0 (0–400)	<0.001
Weight (kg)	66.65 ± 12.23	68.33 ± 11.83	0.400
FEV₁ (L)	2.91 ± 0.72	1.93 ± 0.66	<0.001
FVC (L)	3.62 ± 0.87	3.40 ± 2.14	0.396
FEV₁/FVC (%)	80.53 ± 4.83	59.89 ± 8.19	<0.001
PEF (L/s)	7.44 ± 1.94	4.62 ± 1.73	<0.001
MEF75% (L/s)	6.65 ± 1.85	2.85 ± 1.32	<0.001
MEF50% (L/s)	3.72 ± 1.18	1.27 ± 0.58	<0.001
MEF25% (L/s)	1.12 ± 0.59	0.37 ± 0.15	<0.001
FEF25–75% (L/s)	2.80 ± 1.00	0.96 ± 0.42	<0.001
MVV (L)	100.44 ± 25.87	64.17 ± 22.36	<0.001

Continuous variables are presented as mean ± standard deviation or median (interquartile range), according to data distribution. Categorical variables are presented as number (%). Comparisons were performed between patients with NE-COPD and Non-COPD controls. BMI, body mass index; FEV₁, forced expiratory volume in one second; FVC, forced vital capacity; PEF, peak expiratory flow; MEF75%, maximal expiratory flow at 75% of FVC; MEF50%, maximal expiratory flow at 50% of FVC; MEF25%, maximal expiratory flow at 25% of FVC; FEF25–75%, forced expiratory flow between 25% and 75% of forced vital capacity; MVV, maximal voluntary ventilation; IQR, interquartile range.

### 3.2 CT-derived emphysema burden

**Figure 5:**
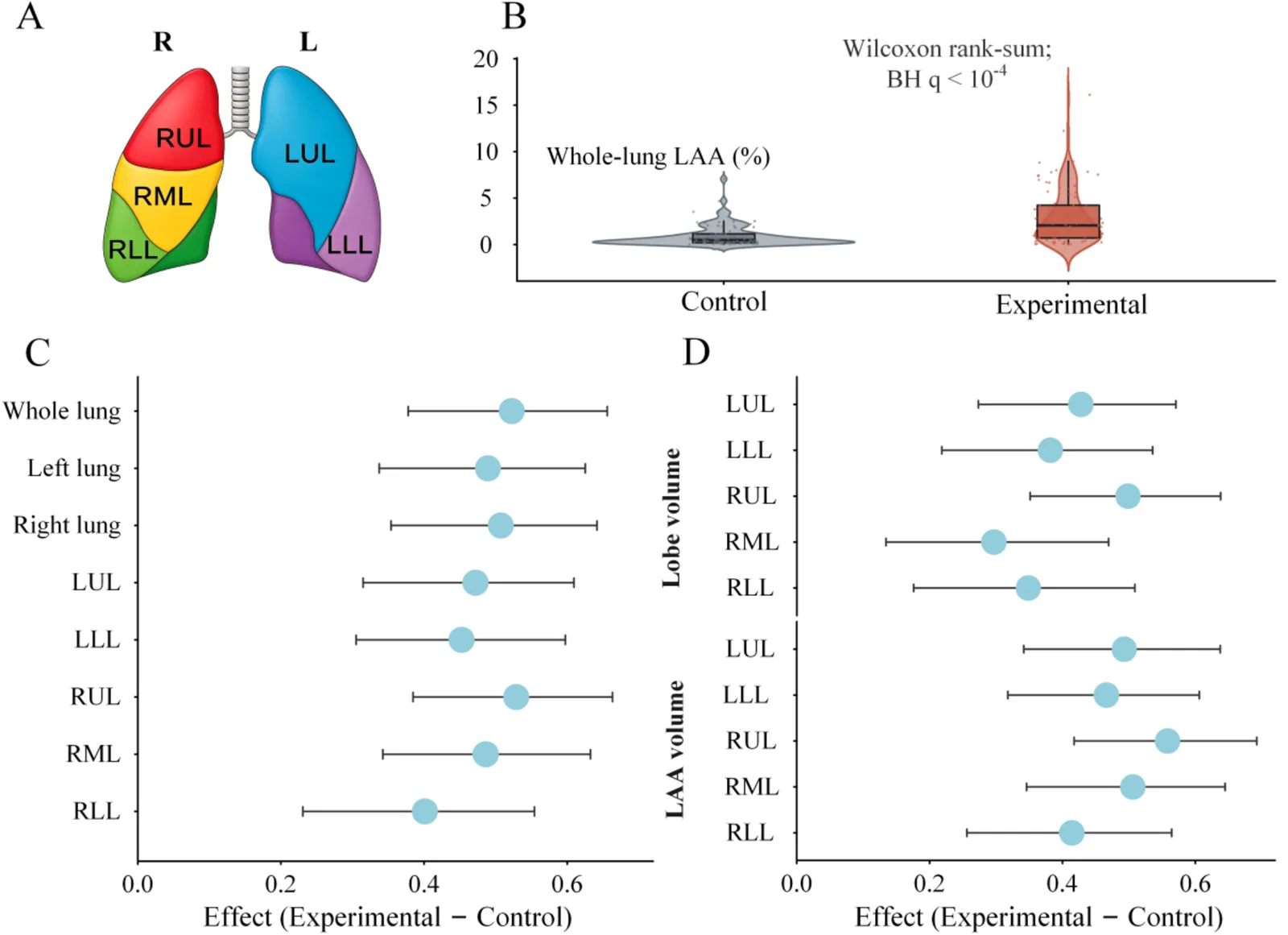
Whole-lung and lobar low-attenuation area burden in the experimental and control groups. **(A)** Schematic lung map showing the median between-group difference in the percentage of low-attenuation area (LAA; attenuation < −950 HU) for each pulmonary lobe. **(B)** Distribution of whole-lung LAA percentages in the control group (n = 86) and experimental group (n = 87). **(C)** Between-group differences in LAA percentage for the whole lung, left and right lungs, and individual lobes. **(D)** Between-group differences in absolute lobe volume and LAA volume for each pulmonary lobe.

We further quantified the low-attenuation area percentage (LAA%-950) to characterize emphysema burden in both groups. Although all NE-COPD participants were defined as having minimal emphysema (LAA%-950 <5%), the distribution of whole-lung LAA percentages was shifted towards higher values in the NE-COPD group compared with Non-COPD controls [median [IQR], 2.058% [0.716%–4.228%] vs. 0.493% [0.192%–1.141%]; median difference, 1.564 percentage points; Cliff's δ = 0.522; Benjamini–Hochberg-adjusted q = 1.46 × 10⁻^8^]. Spatial mapping of lobar LAA percentages showed positive between-group differences in all five lobes, with the largest increase in the right middle lobe and the smallest in the right lower lobe (Figure 5A). Lobe-level median differences ranged from 0.690 to 2.110 percentage points, with Cliff's δ values from 0.401 to 0.529 and all adjusted q-values below 5.6 × 10⁻^6^. Positive effect sizes were also observed for absolute LAA volume and total lobar volume; however, the concordant increases in lobar LAA percentages suggest that the greater LAA burden in the NE-COPD group cannot be attributed solely to differences in lung volume (Figures 5B–D). These findings confirm that, despite the minimal emphysema threshold, the NE-COPD group exhibited a systematically higher low-attenuation burden than the control group, supporting the phenotypic distinction.

### 3.3 CT-derived airway morphometric differences

CT-derived airway morphometric measures differed between patients with NE-COPD and controls. The global k value was less negative in the COPD group than in controls (−1.771 ± 0.431 vs. −1.964 ± 0.339; mean difference, 0.193; standardised mean difference, 0.50; false discovery rate-adjusted *p* = 0.002), indicating altered global airway branching complexity.

Compared with Non-COPD controls, patients with NE-COPD demonstrated significantly altered fourth-generation airway inter-tapering indices in multiple lobes. Specifically, increased lobar inter-tapering values were observed in the right upper lobe, right middle lobe, right lower lobe, and left upper lobe, indicating altered airway calibre transition patterns between proximal parent airways and distal daughter branches. Although several lobar inter-tapering indices differed between groups, only selected features retained after LASSO were included in the final prediction model. Among the evaluated airway metrics, the right middle lobe fourth-generation inter-tapering index showed the largest between-group difference (40.397 ± 9.223 vs. 33.125 ± 7.440; mean difference, 7.272; standardised mean difference, 0.88; false discovery rate-adjusted *p* < 0.001) (Table 2). These findings suggest that regional differences in airway geometric characteristics may be detectable in patients with airflow limitation despite minimal emphysematous destruction.

**Table 2:** CT-derived airway morphometric measures in Non-COPD controls and patients with NE-COPD.

Parameter	Non-COPD (*n* = 86)	NE-COPD (*n* = 87)	Mean difference (COPD−control)	Effect size (SMD)¹	FDR-adjusted *P* (*q* value)²
Global k value	−1.964 ± 0.339	−1.771 ± 0.431	0.193	0.50	0.002
G4 RUL inter-tapering	38.243 ± 6.936	43.438 ± 7.051	5.195	0.75	<0.001
G4 RML inter-tapering	33.125 ± 7.440	40.397 ± 9.223	7.272	0.88	<0.001
G4 RLL inter-tapering	22.780 ± 7.849	26.964 ± 9.282	4.184	0.49	0.002
G4 LUL inter-tapering	35.688 ± 6.543	41.294 ± 7.900	5.606	0.77	<0.001
G4 LLL inter-tapering	31.992 ± 7.844	35.226 ± 7.076	3.234	0.44	0.005

Quantitative airway measurements include fourth-generation lobar inter-tapering indices. Values are presented as mean ± standard deviation or median (interquartile range). Higher inter-tapering values indicate greater calibre reduction from parent to daughter airway branches and reflect altered airway calibre transition patterns. Effect size was calculated using the standardized mean difference (SMD).^2^ *P* values were adjusted using the Benjamini-Hochberg false discovery rate method. FDR, false discovery rate; G4, fourth-generation airway; RUL, right upper lobe; RML, right middle lobe; RLL, right lower lobe; LUL, left upper lobe; LLL, left lower lobe.

### 3.4 Associations between airway metrics and pulmonary function

Airway morphometric measures were associated with airflow-related pulmonary function indices. The global k value showed inverse correlations with peak expiratory flow, FEV₁, and maximal voluntary ventilation, with correlation coefficients of −0.45, −0.43, and −0.42, respectively, all with *p* < 0.001. These associations remained significant after adjustment for age, sex, and smoking exposure. Partial correlation analysis adjusted for age, sex, and smoking exposure was additionally performed.

**Figure 6:**
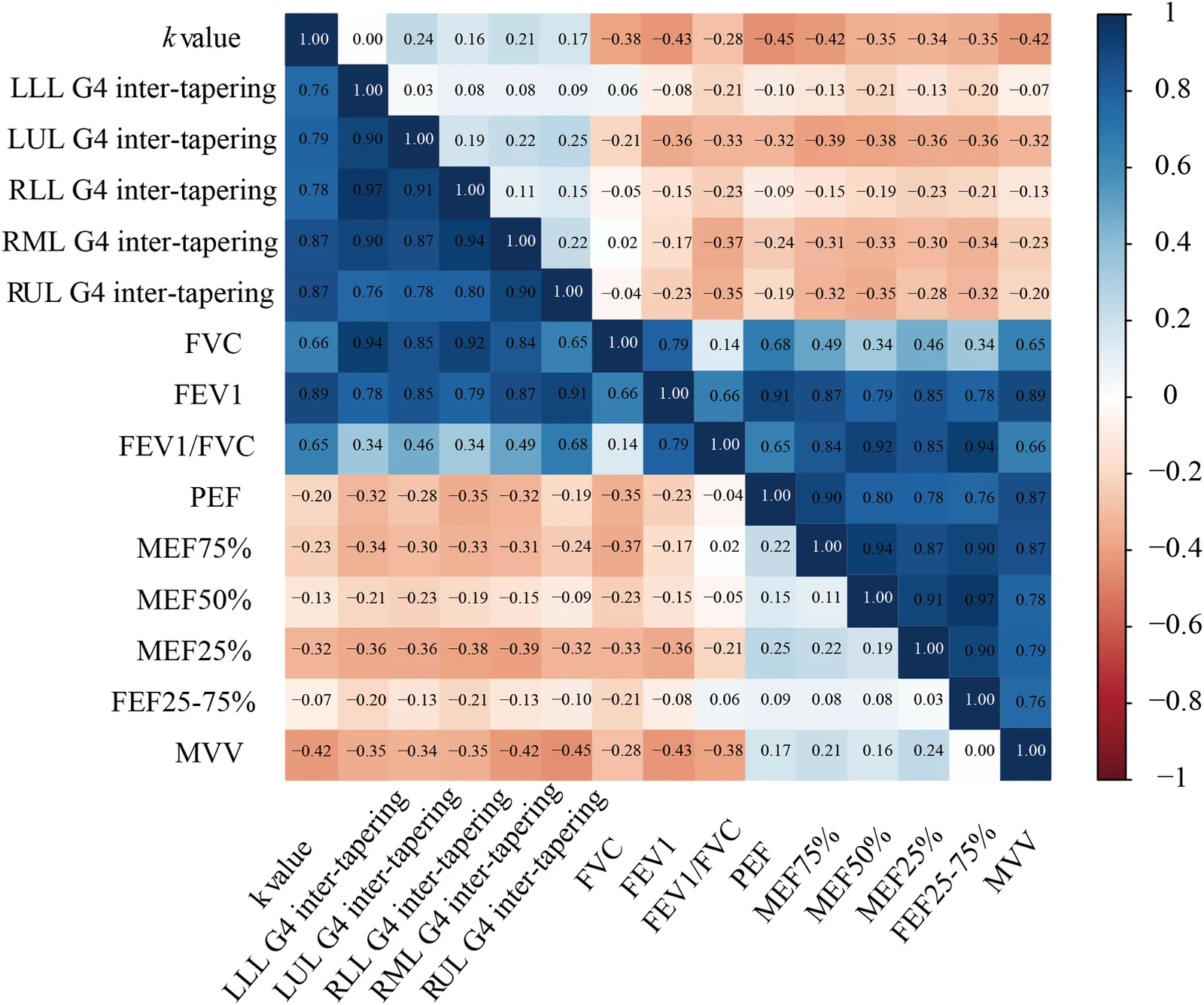
Correlation matrix of pulmonary function indices and CT-derived airway features. G4, fourth-generation airway; RUL, right upper lobe; RML, right middle lobe; RLL, right lower lobe; LUL, left upper lobe; LLL, left lower lobe; FEV₁, forced expiratory volume in one second; FVC, forced vital capacity; PEF, peak expiratory flow; MEF75%, maximal expiratory flow at 75% of FVC; MEF50%, maximal expiratory flow at 50% of FVC; MEF25%, maximal expiratory flow at 25% of FVC; FEF25-75%, forced expiratory flow between 25% and 75% of forced vital capacity; MVV, maximal voluntary ventilation.

Right middle lobe fourth-generation inter-tapering was inversely correlated with FEV₁/FVC and MEF50%, with correlation coefficients of −0.35 and −0.33, respectively, both with *p* < 0.001. Correlations between inter-tapering indices and FVC were generally weaker, with absolute correlation coefficients of 0.20 or less (Figure 6). The strongest correlations were observed between lobar inter-tapering parameters and airflow obstruction indices, suggesting that altered airway geometric characteristics may be related to pulmonary functional impairment.

### 3.5 Construction and performance of predictive models

**Figure 7:**
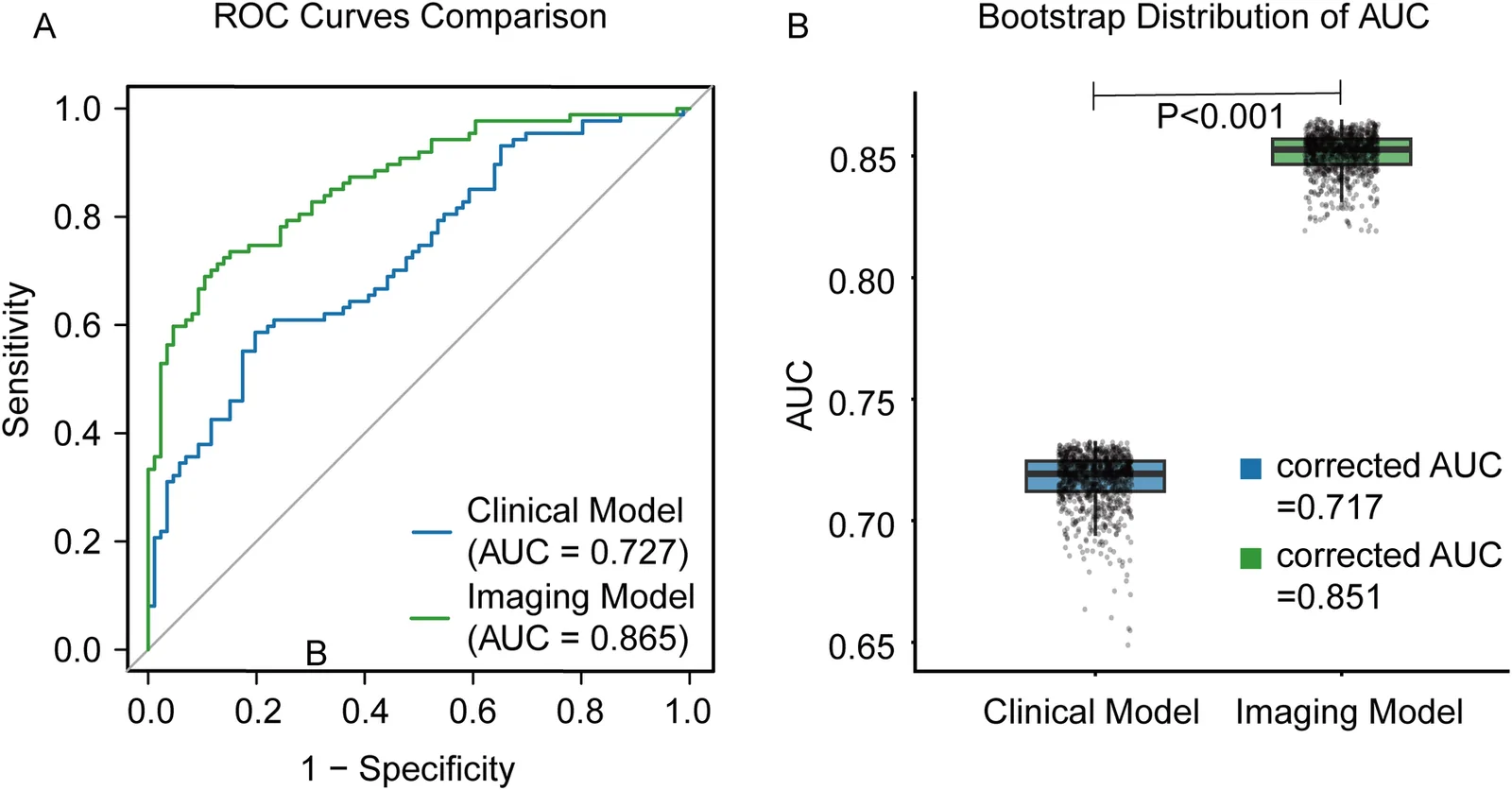
Comparison of predictive performance between clinical and imaging-augmented models. **(A)** Apparent receiver operating characteristic curves of the two models. **(B)** Bootstrap distributions of optimism-corrected AUCs. The optimism-corrected AUCs were 0.717 for the baseline clinical model and 0.851 for the imaging-augmented model.

A clinical prediction model was first constructed using age, sex, and smoking exposure. The clinical model demonstrated moderate discrimination, with an apparent AUC of 0.727. After 1,000 bootstrap resampling iterations, the estimated optimism was 0.010, resulting in an optimism-corrected AUC of 0.717 (95% CI, 0.689–0.730) (Figures 7A,B).

**Figure 8:**
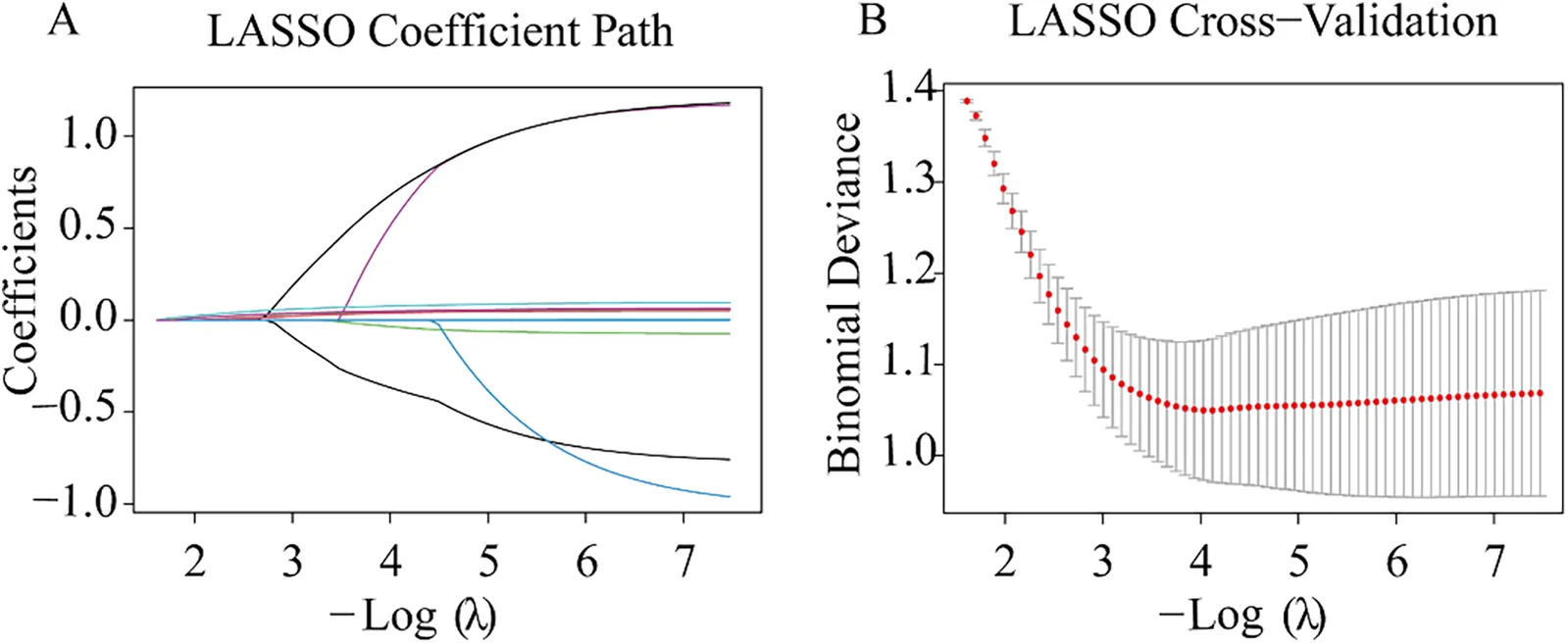
Variable selection and tuning using LASSO. **(A)** Coefficient profiles of candidate predictors across the range of penalty parameters. **(B)** Cross-validation curve for selection of the optimal penalty parameter *λ* based on binomial deviance. The optimal *λ* was selected using the minimum cross-validated deviance criterion. Red dots indicate the mean cross-validated deviance, and grey bars indicate the corresponding standard errors.

LASSO regression was subsequently performed for imaging feature selection. The final imaging-augmented model incorporated the clinical variables together with global airway branching complexity (global k value) and four fourth-generation lobar inter-tapering indices, including G4 LLL, G4 LUL, G4 RML, and G4 RUL inter-tapering (Figure 8). The imaging-augmented model demonstrated improved discrimination compared with the clinical model. The apparent AUC was 0.865, with an estimated bootstrap optimism of 0.014 and an optimism-corrected AUC of 0.851 (95% CI, 0.829–0.863) (Figures 7A,B). The difference in AUC between the two models was statistically significant according to DeLong's test. The complete model coefficients and selected variables are provided in Table 3. These findings indicate that incorporating quantitative airway geometric features improved discrimination within the studied cohort, although the independent incremental value of inter-tapering beyond established quantitative CT descriptors requires further validation.

**Table 3:** Multivariable logistic regression models for predicting non-emphysematous COPD.

Predictor	*β* coefficient	OR	95% CI	*P* value	VIF
Intercept	−10.933	0.000	0.000–0.003	<0.001	NA
Age	0.051	1.052	1.013–1.095	0.0099	1.09
Sex (male)	−0.652	0.521	0.208–1.269	0.156	1.29
Smoking index	0.002	1.002	1.000–1.004	0.052	1.28
Global k value	1.102	3.009	0.959–9.923	0.063	1.21
G4 LLL inter-tapering	0.063	1.065	1.011–1.125	0.021	1.06
G4 LUL inter-tapering	0.055	1.056	0.997–1.122	0.066	1.06
G4 RML inter-tapering	0.097	1.102	1.050–1.161	<0.001	1.09
G4 RUL inter-tapering	0.060	1.062	1.001–1.130	0.051	1.05

The imaging-augmented model was constructed using clinical variables, global airway branching complexity, and LASSO-selected lobar inter-tapering features. The imaging-augmented model included clinical variables (age, sex, and smoking exposure), global airway branching complexity (global k value), and four fourth-generation lobar inter-tapering indices (G4 LLL, G4 LUL, G4 RML, and G4 RUL). Regression coefficients, odds ratios, confidence intervals, and *P* values are provided. NE-COPD, non-emphysematous chronic obstructive pulmonary disease; *OR,* odds ratio; *CI*, confidence interval; VIF, variance inflation factor; G4, fourth-generation airway; LLL, left lower lobe; LUL, left upper lobe; RML, right middle lobe; RUL, right upper lobe.

### 3.6 Model validation and calibration

**Figure 9:**
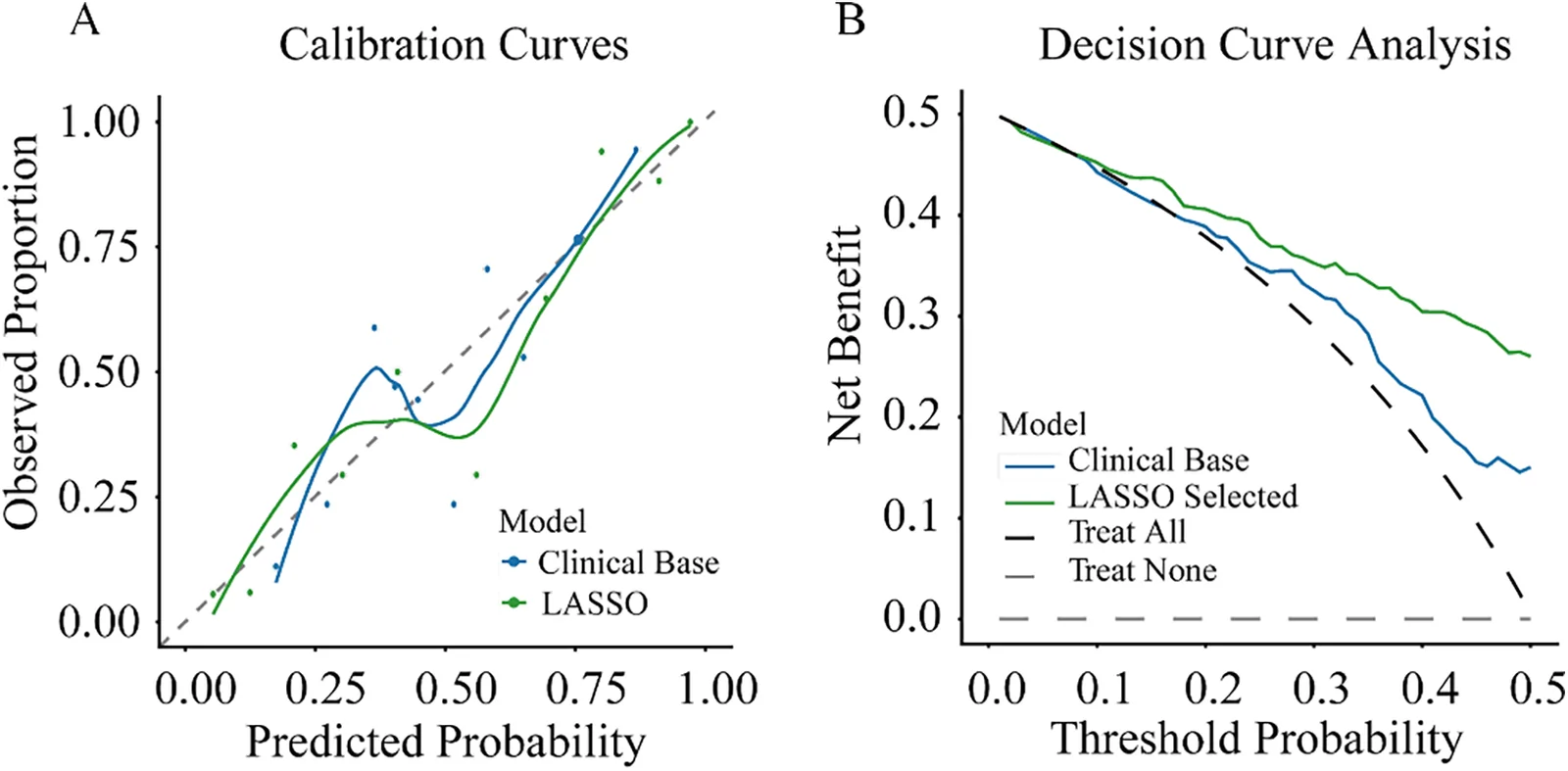
Calibration and clinical utility assessment of the prediction models. **(A)** Calibration curves comparing predicted probabilities with observed proportions for the baseline clinical model and the imaging-augmented model. The dashed diagonal line indicates perfect calibration. **(B)** Decision curve analysis illustrating the potential clinical utility of the baseline clinical model and the imaging-augmented model across a range of threshold probabilities, with the “treat all” and “treat none” strategies shown for reference.

Internal validation was performed using 1,000 bootstrap resampling iterations. For the final imaging-augmented model, the calibration slope was 0.853 (95% CI, 0.615–1.144), and the calibration intercept was −0.009 (95% CI, −0.373–0.355), indicating acceptable calibration after internal validation (Figure 9A).

### 3.7 Decision curve analysis

Decision curve analysis was performed to evaluate the potential clinical utility of the two prediction models. Across the evaluated threshold probability range, the imaging-augmented model demonstrated higher net benefit than the clinical model and alternative strategies. The greatest relative improvement was observed within a threshold probability range of approximately 15%–35% (Figure 9B). This finding indicates that, within the studied cohort, incorporating quantitative airway features may provide additional decision-analytic benefit beyond clinical variables alone. However, these threshold probabilities represent model-based estimates rather than predefined clinical decision thresholds.

### 3.8 Interpretation of imaging feature contribution using SHAP analysis

**Figure 10:**
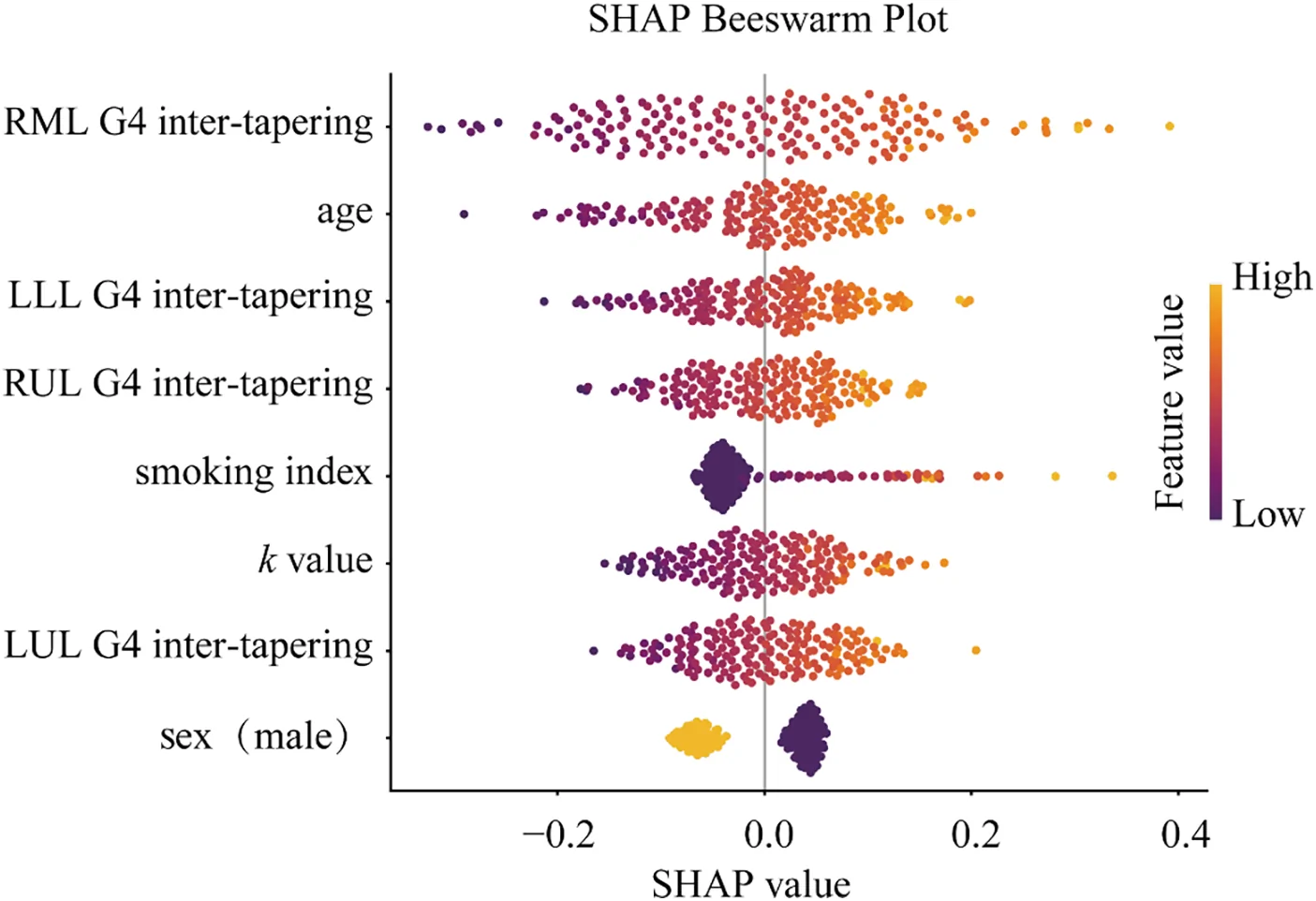
SHAP summary plot of the final imaging-augmented model. SHAP values quantify the contribution of each predictor to individual model predictions. Positive SHAP values indicate increased predicted probability of NE-COPD, whereas negative values indicate decreased predicted probability.

SHAP analysis was performed to evaluate the relative contribution of individual predictors in the imaging-augmented model (Figure 10). Among the selected variables, the four lobar fourth-generation inter-tapering indices collectively accounted for the majority of model contribution. The right middle lobe fourth-generation inter-tapering index showed the highest individual contribution within the current model. However, this finding should be interpreted as a model-specific feature contribution rather than evidence of a unique biological dominance of the right middle lobe. Further validation is required to determine whether regional differences represent true anatomical susceptibility or reflect measurement-related factors.

## 4 Discussion

In this study, we evaluated whether quantitative CT-derived lobar inter-tapering could characterize differences in airway geometry among individuals with non-emphysematous COPD (NE-COPD). We found that fourth-generation lobar inter-tapering differed between participants with airflow limitation and minimal emphysematous destruction and that incorporation of inter-tapering features into a clinical model improved discrimination within the studied cohort. These findings suggest that airway branching geometry may represent an additional imaging dimension associated with airway-predominant COPD characteristics. Importantly, inter-tapering should not be interpreted as a diagnostic replacement for spirometry, an established disease descriptor, or direct evidence of airway remodeling. Rather, it should currently be considered a candidate geometric CT descriptor that requires further evaluation against established quantitative imaging descriptors, physiological measurements, and biological data.

The interpretation of the present findings requires consideration within the broader evolution of quantitative COPD imaging. COPD is increasingly recognized as a heterogeneous disease in which airflow limitation may reflect varying contributions from airway abnormalities, small-airway dysfunction, and parenchymal destruction (1, 3, 4). Large cohort programs, including COPDGene, SPIROMICS, and ECLIPSE, have provided complementary frameworks for characterizing this heterogeneity through clinical, physiological, and imaging measurements (3, 11, 13, 31). In particular, quantitative CT studies arising from these cohorts have characterized partially overlapping dimensions of disease, including emphysema, airway abnormalities, gas trapping, and their relationships with pulmonary function and clinical outcomes. These developments have expanded quantitative COPD imaging from the assessment of individual structural abnormalities toward a more multidimensional characterization of pulmonary structure and function.

Within this multidimensional framework, established quantitative CT descriptors provide information on different aspects of pulmonary structural and functional abnormalities. Airway wall thickness, Pi10, and airway lumen measurements characterize airway wall and luminal dimensions (8, 9, 15), whereas CT-derived airway count has been investigated as a measure of detectable airway loss and has been associated with COPD severity and progression (32). Functional approaches, including expiratory CT-derived gas trapping and parametric response mapping (PRM), provide complementary assessment of regional functional small-airway abnormalities and emphysema (12, 14). These approaches have substantially advanced COPD phenotyping, but each captures a particular dimension of disease. Accordingly, no single quantitative imaging parameter should be expected to represent the full spectrum of structural, functional, and biological heterogeneity in COPD.

Within this evolving framework, the rationale for investigating inter-tapering is that existing quantitative CT descriptors primarily characterize specific aspects of COPD-related abnormalities, including airway wall properties, airway narrowing, airway loss, emphysema burden, and functional small-airway impairment. In contrast, the hierarchical organization of airway branching across generations has received comparatively less attention. Inter-tapering quantifies the relative transition in airway calibre between proximal parent airways and distal daughter branches and therefore describes a geometric property of airway branching organization rather than a single anatomical feature such as wall thickness, lumen size, or airway count. Accordingly, inter-tapering may represent a complementary geometric CT descriptor within the multidimensional framework of COPD imaging. However, the biological interpretation of altered inter-tapering remains uncertain. The present study does not establish whether increased inter-tapering directly reflects airway structural alterations, airway narrowing, airway pruning, developmental variation, or other pathological processes. Therefore, inter-tapering should currently be interpreted as an empirical imaging feature associated with airflow limitation rather than an established marker of a specific biological mechanism.

The clinical model incorporating age, sex, and smoking exposure achieved moderate discrimination for identifying NE-COPD, and the addition of quantitative airway features further improved discrimination within the studied cohort. This improvement should nevertheless be interpreted in the context of established quantitative COPD imaging. COPDGene was designed to integrate epidemiological, genetic, clinical, physiological, and imaging information across a large cohort (11), and subsequent quantitative imaging work has demonstrated that emphysema, airway abnormalities, and other CT-derived features capture related but non-identical aspects of COPD (3, 10). SPIROMICS has similarly established standardized multicenter quantitative CT methods for assessing airway and parenchymal abnormalities (13).

An important unresolved question is whether inter-tapering provides information beyond established quantitative CT descriptors or instead reflects structural or functional abnormalities already captured by existing measures. The present study was not designed to determine its incremental value beyond airway wall thickness, Pi10, airway count, emphysema burden, expiratory gas trapping, or PRM-defined functional small-airway disease. These descriptors characterize different aspects of COPD: airway wall and lumen measurements quantify central airway dimensions, airway count reflects detectable airway loss, and gas trapping and PRM provide information on regional functional small-airway abnormalities. Inter-tapering, by contrast, quantifies relative calibre transition between successive airway generations. It may therefore provide complementary information regarding airway branching geometry, but redundancy with established structural or functional measures cannot be excluded. Future studies incorporating these descriptors within the same population will be required to determine whether inter-tapering provides independent incremental information.

The biological interpretation of altered inter-tapering requires careful consideration. Airway calibre transition is influenced by airway architecture and geometry, but the present study cannot determine whether altered inter-tapering reflects airway narrowing, airway loss or pruning, developmental variation, or other disease-related processes (5–7, 33, 34). Quantitative CT and PRM studies have established complementary structural and functional imaging phenotypes in COPD (12, 14), but these imaging phenotypes should not by themselves be interpreted as direct surrogates of specific biological mechanisms.

Beyond quantitative CT phenotyping, multimodal studies have further illustrated the biological heterogeneity underlying COPD-related structural abnormalities. Hardin et al. demonstrated that asthma-COPD overlap is characterized by distinct clinical, radiographic, and genetic features compared with COPD alone (35). More recently, Fangal et al. identified distinct physiological, transcriptomic, and imaging characteristics across asthma, COPD, and asthma-COPD overlap, illustrating that partially overlapping imaging features may occur in different physiological and molecular contexts (36). Multi-omics analyses by Gillenwater et al. further demonstrated that COPD heterogeneity extends to coordinated transcriptomic, proteomic, metabolomic, and clinical variation (37). Collectively, these findings support interpreting quantitative CT descriptors as components of a multidimensional disease framework rather than as direct indicators of a single pathological mechanism. Within this context, lobar inter-tapering should currently be regarded as a candidate geometric CT descriptor whose biological significance and incremental value beyond established quantitative CT measures remain to be determined through studies integrating structural and functional imaging with complementary physiological and molecular phenotyping.

The potential clinical implication of this study is not that CT-based airway geometric measurements can replace pulmonary function testing or establish a new diagnostic pathway. Instead, quantitative airway geometry analysis may provide an additional structural perspective for individuals with airflow limitation despite minimal emphysematous destruction. Within the broader framework of COPD phenotyping, inter-tapering may complement existing assessments of emphysema, airway morphology, and functional small-airway abnormalities. However, its clinical applicability requires further evaluation, including assessment of reproducibility, external validation, and incremental value beyond established quantitative CT descriptors.

Several limitations should be acknowledged. First, this was a retrospective single-center study with a relatively limited sample size. Although internal validation using bootstrap resampling was performed, external validation in independent multicenter cohorts is necessary to confirm the generalizability of our findings. Second, the control group consisted of individuals who underwent CT and pulmonary function testing for clinical reasons rather than healthy community volunteers. Therefore, selection bias cannot be completely excluded. Third, COPD was defined using the fixed post-bronchodilator FEV₁/FVC threshold of 0.70. Lower limit of normal (LLN)-based classification was not available in our retrospective dataset, and future studies incorporating LLN-based definitions are warranted. Fourth, our analysis relied on inspiratory CT only, and expiratory CT-derived gas trapping or PRM-defined functional small airway disease was not available. Consequently, we could not determine whether altered inter-tapering is associated with functional small airway impairment or represents a purely structural characteristic. Furthermore, established quantitative CT descriptors, including airway wall thickness, Pi10, airway count, and PRM-derived measures, were not simultaneously assessed in the current cohort. Therefore, the present study cannot establish whether inter-tapering provides independent information beyond established structural and functional quantitative CT measures. Future multimodal studies integrating airway morphology, gas trapping, PRM, pulmonary physiology, and biological profiling are required to clarify the specific contribution of inter-tapering within COPD phenotyping. Fifth, although automated airway analysis underwent visual quality control, formal interobserver and intraobserver reproducibility analyses were not prospectively collected. Future studies should include dedicated reproducibility evaluation. Finally, although LASSO regression and bootstrap validation were applied to reduce overfitting risk, the imaging-augmented model requires validation in larger cohorts before clinical application.

In conclusion, quantitative CT-derived lobar inter-tapering was associated with pulmonary function-defined non-emphysematous COPD and improved discrimination beyond basic clinical variables within this exploratory cohort. These findings suggest that lobar inter-tapering represents a candidate geometric CT descriptor reflecting differences in airway branching organization among individuals with non-emphysematous COPD. However, the biological basis and incremental value of inter-tapering beyond established quantitative CT descriptors remain uncertain. Future multicenter studies integrating structural imaging, functional imaging approaches, pulmonary physiology, and biological profiling are required to determine whether inter-tapering provides complementary information within the multidimensional framework of COPD phenotyping.

## Data Availability

The original contributions presented in the study are included in the article/Supplementary Material, further inquiries can be directed to the corresponding author.
